# Supplementary material for: Leveraging machine learning for enhanced and interpretable risk prediction of venous thromboembolism in acute ischemic stroke care
Source: PLoS One. 2025 Mar 18;20(3):e0302676. doi: 10.1371/journal.pone.0302676 (PMC11918378; doi:10.1371/journal.pone.0302676)
Supplement: S1 Table — (DOCX) [file pone.0302676.s001.docx]

**S1 Table1. Raw data integrity investigation.**

| **Column** | **MissingCount** |
| --- | --- |
| gender | 0 |
| Ethnicity | 0 |
| Wake up stroke | 0 |
| Weakness | 0 |
| dysarthria | 0 |
| Other symptoms | 0 |
| Dizziness | 0 |
| Paresthesia | 0 |
| Headache | 0 |
| Vomit | 0 |
| Convulsion | 0 |
| Pre-morbid mRS | 38 |
| ECG | 0 |
| Other comorbid conditions | 0 |
| History of cerebral infarction | 0 |
| Atrial fibrillation | 0 |
| DM | 0 |
| Prehospital medication | 0 |
| Thrombolytic therapy | 0 |
| EVT | 0 |
| Smoking | 0 |
| Drinking | 0 |
| mRS after admission | 38 |
| Aspirin | 0 |
| Clopidogrel | 0 |
| Heparin | 0 |
| Enoxaparin | 0 |
| Warfarin | 0 |
| Rivaroxaban | 0 |
| Sulfonylureas | 0 |
| Glycosidase inhibitor | 0 |
| Biguanides | 0 |
| Anti-infective treatment | 0 |
| Chinese medicines during hospitalization | 0 |
| Lipid medicine | 0 |
| Antiplatelet therapy during hospitalization | 0 |
| Anticoagulant therapy during hospitalization | 0 |
| Antilipidemic drugs during hospitalization | 0 |
| Antidiabetic treatment during hospitalization | 0 |
| Anti-infective treatment | 0 |
| Large vessel occlusion | 0 |
| Small vessel occlusive stroke | 0 |
| Cardioembolic stroke | 0 |
| Other causes of stroke | 0 |
| Unexplained stroke | 0 |
| Age | 0 |
| GCS | 21 |
| SBP | 1 |
| DBP | 1 |
| Height | 0 |
| Weight | 0 |
| NIHSS after admission | 29 |
| White_blood_cell_count | 51 |
| Neutrophil count | 53 |
| Lymphocyte count | 53 |
| hsCRP | 325 |
| INR | 102 |
| Fibrinogen | 106 |
| D-dimer | 151 |
| Alanine aminotransferase | 104 |
| LDLC | 135 |
| NIHSS onset | 0 |
