# Supplementary material for: Leveraging machine learning for enhanced and interpretable risk prediction of venous thromboembolism in acute ischemic stroke care
Source: PLoS One. 2025 Mar 18;20(3):e0302676. doi: 10.1371/journal.pone.0302676 (PMC11918378; doi:10.1371/journal.pone.0302676)
Supplement: S2 Table — (DOCX) [file pone.0302676.s002.docx]

| **Variable** | **Category** | **OR** | **OR(95%CI)** | **P-value** |
| --- | --- | --- | --- | --- |
| heparin | Yes vs No | 16.23 | 3.91-67.35 | <0.001 |
| Antiinfectivetreatment | Yes vs No | 6.57 | 2.26-19.03 | 0.001 |
| Anticoagulanttherapyduringhospitalization | Yes vs No | 5.88 | 2.11-16.404 | 0.001 |
| Chinesemedicinesduringhospitalization | Yes vs No | 27.74 | 6.60-116.58 | <0.001 |
| unexplainedstroke | Yes vs No | 11.27 | 3.64-34.82 | <0.001 |
|  |  |  |  | 0.002 |
| NIHSSonset | 5-14 vs 0-4 | 1.84 | 0.15-21.92 | 0.629 |
|  | 15-20 vs 0-4 | 16.37 | 1.82-146.84 | 0.013 |
|  | 21-42 vs 13-15 | 9.19 | 1.05-80.05 | 0.045 |
| GCS | 3-8 vs 13-15 | 5.59 | 1.24-25.09 | 0.025 |
|  | 9-12 vs 13-15 | 0.32 | 0.04-2.19 | 0.244 |
| Ddimer |  | 1 | 1-1.01 | 0.003 |
| hsCRP |  | 1.02 | 1.01-1.03 | 0.024 |

**S2 Table. Stepwise forward logistic regression multivariate analysis.**
