# Supplementary figures and images for: Leveraging machine learning for enhanced and interpretable risk prediction of venous thromboembolism in acute ischemic stroke care
Source: PLoS One. 2025 Mar 18;20(3):e0302676. doi: 10.1371/journal.pone.0302676 (PMC11918378; doi:10.1371/journal.pone.0302676)

# Precision-Recall Curve

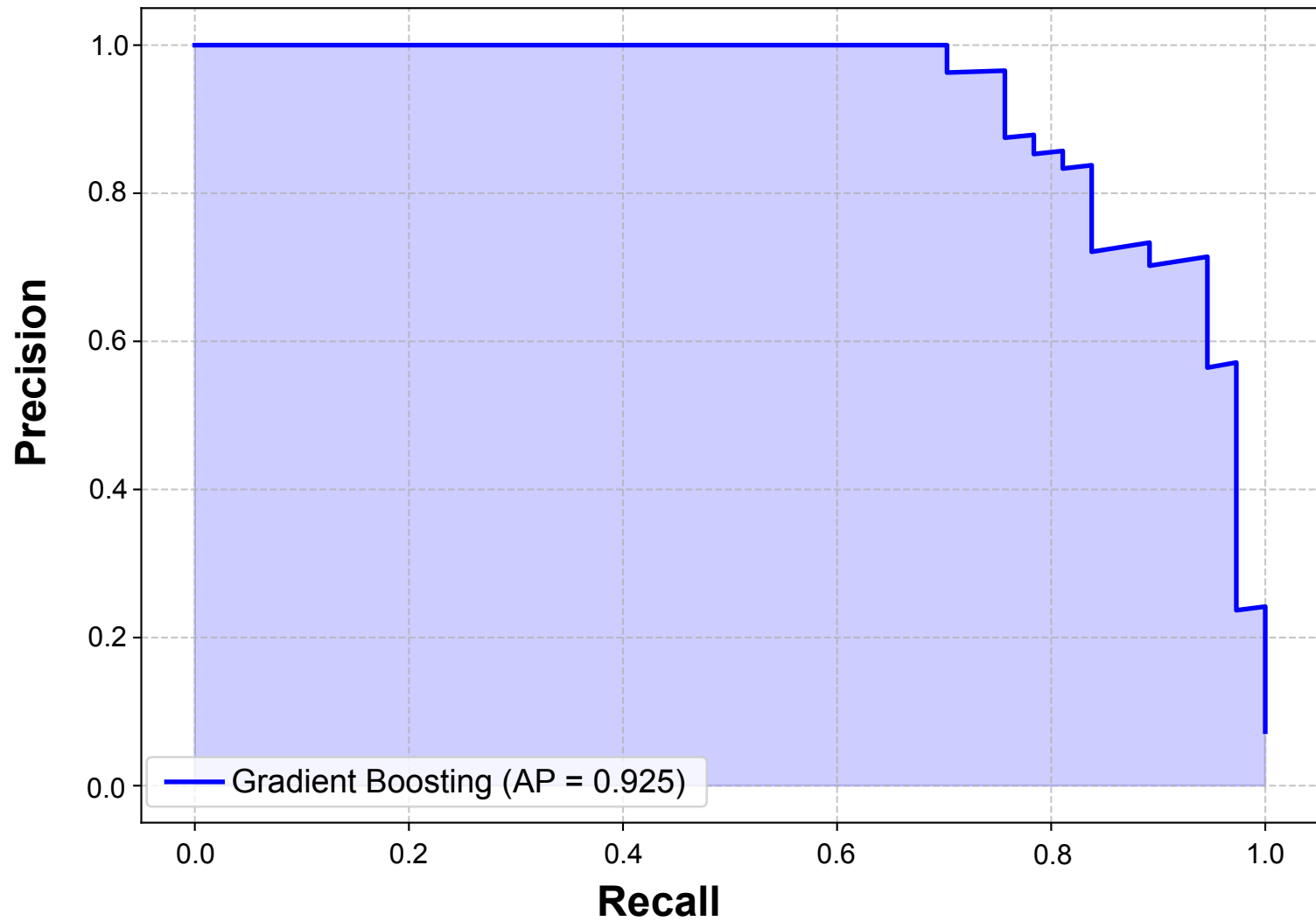

Supplement: S4 File — (PDF) [file pone.0302676.s004.pdf]
