# Supplementary material for: Leveraging machine learning for enhanced and interpretable risk prediction of venous thromboembolism in acute ischemic stroke care
Source: PLoS One. 2025 Mar 18;20(3):e0302676. doi: 10.1371/journal.pone.0302676 (PMC11918378; doi:10.1371/journal.pone.0302676)
Supplement: Attachment — Submitted filename: plos one 20240604.docx [file pone.0302676.s005.docx]

The manuscript aims to present an innovative, machine learning-based approach to predict VTE risk in acute ischemic stroke patients. However, there are some shortcomings in the study design, specific details, and innovation of this article:

1. The existing risk prediction models for deep vein thrombosis (DVT) in patients with acute ischemic stroke are numerous, yet the manuscript fails to emphasize the distinctive features of the study, thus lacking novelty.

2. The Introduction section fails to introduce the existing risk prediction models for venous thromboembolism (VTE) in acute ischemic stroke.

3. The article mentions the use of Synthetic Minority Over-sampling Technique (SMOTE) to augment the dataset. It remains unclear whether SMOTE was applied directly to the entire dataset or solely to the training set. Employing SMOTE on the entire dataset could lead to data leakage, with the presence of synthetic data in the test set undermining the model's genuine effectiveness.

4. Although a series of machine learning algorithms were employed in the study, there is no mention of the principles governing parameter selection.

5. The model construction incorporates stepwise forward logistic regression and LASSO variable selection. However, it is not specified whether these procedures were executed solely on the training set or on the entire dataset.

6. In the development and validation of the predictive model, the performance of seven different machine learning models was evaluated, yet confidence intervals were not reported. It is advisable to supplement this information and conduct Delong's test to compare the AUC values of each model for significant differences.

7. A comparison between this study and existing literature utilizing logistic regression models is provided; however, a deeper discussion on the disparities between these two methodologies is warranted, elucidating why machine learning approaches are more suitable for the study's objectives than traditional logistic regression methods.

8. The study introduces a novel machine learning model exhibiting excellent predictive performance. However, the manuscript fails to contrast it with similar previous studies.

9. The Discussion section lacks a discussion of the study's limitations and should propose future research directions.
