# Supplementary material for: Leveraging machine learning for enhanced and interpretable risk prediction of venous thromboembolism in acute ischemic stroke care
Source: PLoS One. 2025 Mar 18;20(3):e0302676. doi: 10.1371/journal.pone.0302676 (PMC11918378; doi:10.1371/journal.pone.0302676)
Supplement: Attachment — Submitted filename: Response to Reviewers.docx [file pone.0302676.s006.docx]

**Dear Editor and Reviewers,**

Thank you for your all valuable feedback on our manuscript titled “Leveraging Machine Learning for Enhanced and Interpretable Risk Prediction of Venous Thromboembolism in Acute Ischemic Stroke Care.” We have carefully considered each of the reviewers’ and editorial suggestions, and we have made the necessary revisions to address the points raised.

Please note that all changes made in the manuscript are marked in blue font for easy reference. We have provided a detailed point-by-point response in the accompanying rebuttal letter to explain how each concern has been addressed or provide a rationale where applicable.

We hope that the revised manuscript meets the expectations and standards of PLOS ONE and look forward to your further assessment.

Thank you once again for the opportunity to improve our manuscript.

**ACADEMIC EDITOR**

**1. The manuscript lacks a clear articulation of why machine learning is particularly suited for improving VTE risk prediction in AIS patients compared to traditional statistical models. Please enhance the discussion on the specific limitations of existing models and how your approach addresses these gaps.**

**Response:** We will enhance the discussion by elaborating on why machine learning, specifically GBM, is better suited for VTE risk prediction in AIS patients than traditional statistical models. Unlike logistic regression, which assumes linear relationships between variables, machine learning models can capture complex nonlinear interactions between clinical and demographic factors. Our approach addresses limitations in traditional models by uncovering hidden patterns in the data that improve predictive accuracy and clinical decision-making, as evidenced by the AUC of 0.923. Additionally, the use of SHAP values allows for transparent interpretation of variable importance, further supporting clinical utility.

2. **The current literature review does not sufficiently engage with previous studies on VTE risk prediction in stroke patients. A more comprehensive and critical review of the literature is needed, highlighting how your study builds on or differs from existing work.**

**Response:** We acknowledge the need for a more thorough review of previous studies. We will expand our literature review to critically engage with existing work on VTE risk prediction in stroke patients, such as Pan et al.’s logistic regression model (AUC = 0.785) and Bonkhoff et al.’s L1 regularized logit model (AUC = 0.730). By comparing these traditional models to our machine learning approach, we will demonstrate how our model not only improves accuracy but also incorporates novel predictive factors like EVT and prehospital mRS, which were previously overlooked in the literature (Pan et al., 2018), (Bonkhoff et al., 2019). This will clarify how our study builds on and advances prior work.

**3. The manuscript would benefit from a more detailed description of the cohort, including the inclusion and exclusion criteria, demographic details, and the data collection period. This information is crucial for understanding the applicability and limitations of your findings.**

**Response：**Thank you for the insightful suggestion. We have expanded the manuscript to provide a more detailed description of the cohort, including the inclusion and exclusion criteria, demographic details, and data collection period (December 2021 to December 2023). This ensures a clearer understanding of the applicability and limitations of our findings.

**4. The manuscript mentions the use of advanced techniques such as K-nearest neighbor and SMOTE but lacks sufficient detail on data preprocessing and the handling of potential biases introduced by these techniques. Further elaboration on your model development process, including hyperparameter tuning and the selection criteria for the final model, is necessary.**

**Response：**We appreciate the valuable feedback regarding the need for more details on data preprocessing. In response, we have elaborated on how K-nearest neighbor (KNN) imputation was used for missing values and clarified that SMOTE was applied only to the training set to prevent data leakage. Additionally, we have included information on hyperparameter tuning via grid search and provided details on the criteria used for final model selection.

**5. While SHAP values are used for feature importance, the process of feature selection and engineering should be more thoroughly explained, including how domain knowledge (how these features contributed to the model's predictions) was incorporated.**

**Response：**Thank you for your insightful comment regarding feature selection. We have expanded our explanation to detail how domain knowledge was integrated into the process, ensuring clinically relevant features were prioritized. Additionally, we elaborated on the role SHAP values played, not only in determining feature importance but also in guiding the feature engineering process.

**6. The manuscript currently reports AUC as the primary performance metric. To provide a more comprehensive evaluation, please include additional metrics such as sensitivity, specificity, PPV, NPV, and calibration plots. Furthermore, discussion on the potential for overfitting and steps taken to mitigate this should be included. The study's sample size (1,632 participants) is relatively small for machine learning studies aimed at clinical predictions. Please explain (?).**

**Response：**We appreciate the thoughtful suggestion about external validation. Although we were unable to conduct validation using an independent dataset due to data constraints, we have included a detailed discussion on the generalizability of our model. We emphasized the importance of future validation in different populations and clinical settings to ensure wider applicability.

**7. The manuscript mentions that multiple machine learning models were developed, with the Gradient Boosting Machine (GBM) showing the highest AUC. However, it does not provide information about external validation or the use of an independent test set. Without external validation, the generalizability of the model to other populations remains uncertain. If possible, please conduct validation on an independent dataset or provide a discussion on the generalizability of your model.**

**Response:** Thank you for your insightful suggestion regarding the importance of external validation. While we acknowledge the value of validating our model on an independent dataset to enhance its generalizability, we were unable to access such a dataset at this stage of the research. However, we took several measures to strengthen the model's robustness and reduce overfitting. This included rigorous internal validation using a separate test set and cross-validation techniques during model development.

In future research, we plan to collaborate with other institutions to obtain external datasets for further validation. Additionally, we have included a discussion on the potential limitations regarding generalizability in the manuscript to emphasize the importance of external validation and to highlight this as an avenue for future work.

8. **The manuscript mentions IRB approval but lacks detailed information on patient consent and data privacy. Please address.**

**Response：** Thank you for the valuable feedback. We have clarified that, although ethics approval was obtained, patient data was fully anonymized and informed consent was waived due to the retrospective nature of the study. Additionally, we provided further details on how data privacy was maintained, ensuring that all personal identifiers were removed before analysis.

**9. Authors should better articulate how the proposed model would be implemented in or improve clinical practice. A detailed comparison with existing risk scores like the Wells or Caprini scores is needed to contextualize the added value of your model.**

**Response：**We are grateful for the insightful suggestion to compare our model with existing risk scores like Wells and Caprini. In response, we have provided a detailed comparison in the manuscript, highlighting how our model offers advantages in terms of incorporating a wider range of stroke-specific variables and yielding better.

**10. More information is needed about the patient population, including demographics, stroke subtypes, and comorbidities. This will help assess the model's generalizability across different clinical settings.**

**Response：**Thank you for your valuable feedback on the need for more demographic and clinical details. In response, we have expanded the manuscript to include a comprehensive breakdown of the patient population, including demographics, stroke subtypes, and relevant comorbidities. This additional information ensures that readers can better assess the model's generalizability across different clinical settings.

**11. While your model's AUC is highlighted, the clinical implications of these results are not adequately discussed. We recommend providing a clearer interpretation of the model's predictions, including potential benefits and risks.**

**Response：**We appreciate the thoughtful comment regarding the need for a clearer interpretation of the model's clinical implications. In response, we have expanded the discussion to provide a more in-depth explanation of the model's predictions, including both potential benefits and risks. Specifically, we highlight how early identification of high-risk patients can lead to timely interventions, while also acknowledging the risks of over-reliance on model outputs and the potential for false positives. This provides a balanced perspective on the model's clinical utility.

**12. Please address how the model would be integrated into existing clinical workflows. This includes discussing the protocols for managing patients identified as high risk and the potential impact on treatment decisions.**

**Response：**Thank you for the insightful suggestion about integrating the model into clinical workflows. We have now addressed this in the manuscript by discussing how the model could be implemented in practice. We outline protocols for managing patients identified as high-risk, such as the use of prophylactic anticoagulation or mechanical thromboprophylaxis, and discuss the potential impact on treatment decisions. Additionally, we have considered how the model could be incorporated into existing clinical systems, such as electronic health records (EHR), to streamline its use in routine care.

**13. Consider discussing the ethical implications of implementing the model, including the risk of harm from false positives or negatives.**

**Response:**Thank you for the valuable suggestion regarding the ethical implications of implementing our predictive model, particularly concerning the risks associated with false positives and negatives. Our revised manuscript now includes a discussion of these aspects. Specifically, we acknowledge that while the model exhibits high sensitivity (90.83%) and specificity (93.83%), no model is infallible. We emphasize the importance of integrating the model's predictions with clinical judgment and continuously monitoring its performance to mitigate potential harms. This ensures the model’s application enhances clinical outcomes while maintaining patient safety.

**14. The manuscript's abstract is somewhat dense, and the terminology used (e.g., "utilizing advanced technologies," "innovative approach") is vague. This could make it difficult for readers to quickly grasp the key points of the study.**

**Response:** Thank you for your valuable feedback. In response to the suggestion regarding the abstract, we have refined the terminology to eliminate vague expressions and ensure clarity. The abstract now clearly outlines the methods, results, and significance of the study, making it easier for readers to quickly understand the key points. This revision also reflects the updated version of the manuscript. We appreciate your input and believe the changes enhance the communication of the study's findings.

**15. The manuscript's terminology is not consistently precise (e.g., the use of "key predictors" without defining them clearly), which could lead to confusion. The writing should be more concise and the terms better defined to ensure clear communication of the study's findings and implications.**

**Response:** Thank you for your feedback. We have reviewed the manuscript and addressed the issue of unclear terminology, particularly the use of "key predictors." We have now clearly defined terms such as D-dimer levels, pre-morbid mRS, and large vessel occlusion to ensure clarity. The writing has also been refined for conciseness, avoiding vague language and ensuring precise communication of the study’s findings. We believe these revisions enhance the overall clarity and alignment with your suggestion.

**Academic Editor**

**1. Please ensure that your manuscript meets PLOS ONE's style requirements, including those for file naming. The PLOS ONE style templates can be found at**

**https://journals.plos.org/plosone/s/file?id=wjVg/PLOSOne_formatting_sample_main_body.pdf and**

**<https://journals.plos.org/plosone/s/file?id=ba62/PLOSOne_formatting_sample_title_authors_affiliations.pdf>**

**Response:** We have ensured that our manuscript and supplementary files meet PLOS ONE's style requirements, including correct file naming. We have also followed the PLOS ONE style templates for formatting the main body and author information. Please let us know if any further adjustments are needed.

2. **Please note that PLOS ONE has specific guidelines on code sharing for submissions in which author-generated code underpins the findings in the manuscript. In these cases, all author-generated code must be made available without restrictions upon publication of the work. Please review our guidelines at https://journals.plos.org/plosone/s/materials-and-software-sharing#loc-sharing-code and ensure that your code is shared in a way that follows best practice and facilitates reproducibility and reuse.**

**Response:** Thank you for your reminder. We have noted the specific request regarding code sharing. The relevant code will be uploaded as supplementary material to ensure compliance with best practices and promote reproducibility and reuse of research.

**3. Thank you for stating the following financial disclosure:**

**"This study was funded by the High Level Project of Medicine in Longhua, ShenZhen under grant number HLPM201907020102 and construction funds of key medical disciplines in Longhua District, Shenzhen under grant number MKD202007090208."**

**Please state what role the funders took in the study.  If the funders had no role, please state: ""The funders had no role in study design, data collection and analysis, decision to publish, or preparation of the manuscript.""**

**If this statement is not correct you must amend it as needed.**

**Please include this amended Role of Funder statement in your cover letter; we will change the online submission form on your behalf.**

**Response：**Thank you for your guidance regarding the funding statement. I have amended the funding information as requested. The correct grant is from The Scientific Research Projects of Medical and Health Institutions of Longhua District, Shenzhen under grant number 2024052. Youli Jiang, the first author and funder, was actively involved in all key aspects of the study, including study design, data collection, model construction, analysis, and preparation of the manuscript.

**4. In the online submission form, you indicated that "All data files related to this study can be obtained from the inquiry email 66327285@qq.com(Qingshi Zhao)."**

**All PLOS journals now require all data underlying the findings described in their manuscript to be freely available to other researchers, either 1. In a public repository, 2. Within the manuscript itself, or 3. Uploaded as supplementary information.**

**This policy applies to all data except where public deposition would breach compliance with the protocol approved by your research ethics board. If your data cannot be made publicly available for ethical or legal reasons (e.g., public availability would compromise patient privacy), please explain your reasons on resubmission and your exemption request will be escalated for approval.**

**Response：**Thank you for your comments regarding the data availability. The dataset contains sensitive patient information, and public availability would compromise patient privacy, in compliance with the protocol approved by our research ethics board. Therefore, we are unable to publicly share the data in a repository. However, researchers interested in accessing the data can contact the corresponding author at 66327285@qq.com (Qingshi Zhao) for inquiries, and data access will be granted under specific terms that ensure patient confidentiality. We request an exemption based on ethical and privacy concerns.

**5. Please include captions for your Supporting Information files at the end of your manuscript, and update any in-text citations to match accordingly. Please see our Supporting Information guidelines for more information: http://journals.plos.org/plosone/s/supporting-information.**

**Response:** Thank you for your guidance. We were unable to navigate to the specified link; however, we have referred to several recent publications from 2024 in PLOS ONE and have updated the Supporting Information section in accordance with the journal's guidelines. All supporting file captions have been included at the end of the manuscript, and the in-text citations have been updated to align with the requested format. We believe this meets the required standards.

**Review Comments to the Author**

**Please use the space provided to explain your answers to the questions above. You may also include additional comments for the author, including concerns about dual publication, research ethics, or publication ethics. (Please upload your review as an attachment if it exceeds 20,000 characters)**

**Reviewer #1:**

1. **The existing risk prediction models for deep vein thrombosis (DVT) in patients with acute ischemic stroke are numerous, yet the manuscript fails to emphasize the distinctive features of the study, thus lacking novelty.**

**Response：**Thank you for the valuable feedback. We have revised the manuscript to more clearly articulate the study's novelty and significance. In addition to incorporating new predictive factors like EVT and prehospital mRS, we have emphasized the model's superior performance in terms of sensitivity and specificity. Our model demonstrates excellent capability in accurately identifying both false negatives and false positives, which sets it apart from existing models and highlights its clinical relevance in improving VTE risk prediction for AIS patients.

2. **The Introduction section fails to introduce the existing risk prediction models for venous thromboembolism (VTE) in acute ischemic stroke.**

**Response:** Thank you for your suggestion. In response, we have revised the Introduction to emphasize the limitations of current VTE risk prediction models in AIS patients. Specifically, we discussed how conventional tools like the Caprini and Padua scores fail to account for stroke-specific factors, leading to reduced accuracy. Existing models also overlook dynamic and non-linear interactions among clinical variables, which are crucial in predicting VTE in AIS patients. Our machine learning-based model addresses these gaps by incorporating comprehensive stroke-specific factors and using advanced interpretability techniques, offering enhanced predictive accuracy and clinical relevance.

3. **The article mentions the use of Synthetic Minority Over-sampling Technique (SMOTE) to augment the dataset. It remains unclear whether SMOTE was applied directly to the entire dataset or solely to the training set. Employing SMOTE on the entire dataset could lead to data leakage, with the presence of synthetic data in the test set undermining the model's genuine effectiveness.**

**Response：**Thank you for your insightful comment regarding the application of SMOTE. We appreciate the concern about data leakage. In the revised manuscript, we have clarified that SMOTE was applied only to the training set and not to the entire dataset. This ensured that synthetic data did not contaminate the test set, thus preserving the model’s genuine effectiveness. We have updated the "Data Processing" section to explicitly mention this procedure to avoid any ambiguity.

4. **Although a series of machine learning algorithms were employed in the study, there is no mention of the principles governing parameter selection.**

**Response：**We appreciate your valuable observation regarding the execution of stepwise forward logistic regression and LASSO. In response, we have clarified in the manuscript that both variable selection procedures were performed solely on the training set. This ensures that the test set remains untouched during model training, thereby preventing any bias or overfitting. We have added this clarification to the relevant sections to improve transparency.

5. **The model construction incorporates stepwise forward logistic regression and LASSO variable selection. However, it is not specified whether these procedures were executed solely on the training set or on the entire dataset.**

**Response：**Thank you for the suggestion to provide a deeper discussion on the differences between machine learning and logistic regression. We have now expanded this section to elucidate why machine learning approaches, particularly Gradient Boosting Machine (GBM), are more suitable for our study's objectives. In particular, we explain how machine learning methods better capture complex, nonlinear interactions between variables, which logistic regression often struggles with due to its linear assumptions. This section now contrasts the strengths and limitations of each approach, offering a more thorough justification for using machine learning.

6. **In the development and validation of the predictive model, the performance of seven different machine learning models was evaluated, yet confidence intervals were not reported. It is advisable to supplement this information and conduct Delong's test to compare the AUC values of each model for significant differences.**

**Response：**We appreciate the insightful comment regarding the comparison with similar studies. We have now included a detailed comparison of our model with previous machine learning and logistic regression models from the literature. We highlight that our model not only achieves higher predictive accuracy (AUC of 0.923) but also incorporates SHAP analysis to improve interpretability. This comparison emphasizes the novelty and strengths of our approach in contrast to previous studies.

7. **A comparison between this study and existing literature utilizing logistic regression models is provided; however, a deeper discussion on the disparities between these two methodologies is warranted, elucidating why machine learning approaches are more suitable for the study's objectives than traditional logistic regression methods.**

**Response:** We sincerely appreciate the reviewer’s insightful suggestion. In response, we have expanded the Discussion to provide a deeper comparison between machine learning and logistic regression models. While logistic regression assumes linear relationships, limiting its ability to capture complex, nonlinear interactions, machine learning models such as GBM can model these interactions more effectively, leading to improved predictive accuracy. For instance, our GBM model achieved a higher AUC (0.923) compared to logistic regression models from prior studies (e.g., Pan et al., AUC = 0.785; Bonkhoff et al., AUC = 0.730). Additionally, we enhanced the interpretability of our model using SHAP analysis, allowing clinicians to understand the contribution of each predictor to the overall risk score, something that logistic regression models often lack. This expanded discussion clarifies the suitability of machine learning for our study’s objectives and addresses the reviewer's concerns.

8. **The study introduces a novel machine learning model exhibiting excellent predictive performance. However, the manuscript fails to contrast it with similar previous studies.**

**Response:** We appreciate the feedback and will address this by including a direct comparison with similar studies. Specifically, our GBM model’s performance will be contrasted with recent machine learning models such as Wang et al.‘s support vector machine (AUC = 0.85) for VTE prediction in medical inpatients and Li et al.‘s random forest model (AUC = 0.81) for stroke patients. Our model’s superior AUC of 0.923 and the use of SHAP analysis for interpretability demonstrate both its predictive accuracy and clinical applicability, surpassing these existing approaches (Wang et al., 2019), (Li et al., 2020). We will clearly highlight these comparisons in the manuscript to contextualize our model’s novelty and effectiveness.

**9.The Discussion section lacks a discussion of the study's limitations and should propose future research directions.**

**Response:** Thank you for the suggestion. We have added a discussion of the study’s limitations, such as potential data biases, and proposed future research directions, including external validation and the exploration of additional clinical variables.

**Reviewer #2:**

**The researchers employed machine learning algorithms to enhance the prediction of venous thromboembolism (VTE) risk in patients with acute ischemic stroke. VTE significantly impacts the survival prognosis of stroke patients, and accurate, individualized prediction could further improve patient outcomes. However, I believe there are several areas that need improvement in this study:**

1. **The references in the manuscript are overly concentrated, with multiple studies frequently cited in a single instance. Meanwhile, certain sections of the manuscript that require citations lack the corresponding references.**

**Response:**Thank you for the feedback. We have redistributed the citations to ensure they are more evenly spread across the manuscript, reducing concentration, and have added missing citations where needed to support the claims.

2. **In the “Data processing and feature selection” section, the researchers stated that they used the SMOTE oversampling technique, which is based on the KNN algorithm. References should be added to this section.**

**Response：**Thank you for the suggestion to add references regarding SMOTE and the KNN algorithm. In the revised manuscript, we have included the necessary citations in the “Data Processing and Feature Selection” section. These references now provide clear documentation for the techniques used, ensuring that readers have appropriate context and sources for further reading.

3. **Since SMOTE generates synthetic data, this could significantly affect the model's results. The correct approach is to first split the data into training and test sets, apply SMOTE only to the training set, and leave the test set unprocessed to reduce the risk of overfitting.**

**Response：**We greatly appreciate your valuable observation on the proper application of SMOTE. In line with your suggestion, we have clarified in the manuscript that SMOTE was applied only to the training set and not to the entire dataset. The test set remained unprocessed to ensure a proper evaluation of the model’s performance, thus minimizing the risk of overfitting. This clarification is now explicitly stated in the manuscript, and we have provided additional details to ensure this important step is well-understood.

4. **The authors did not provide a detailed explanation of the hyperparameter tuning process for the machine learning algorithms. In machine learning, model evaluation is typically conducted using a validation set to find the optimal parameters. The researchers did not indicate whether a validation set was used.**

**Response：**We sincerely appreciate this insightful comment. In response, we have clarified the hyperparameter tuning process in the revised manuscript. Specifically, we now explain that grid search was employed within a ten-fold cross-validation framework to optimize the hyperparameters for each machine learning model. Each fold acted as a validation set, ensuring that all model parameters were fine-tuned to prevent overfitting and enhance performance. This process guarantees that the optimal hyperparameters are identified using validation data during cross-validation, which allows for a more accurate evaluation of the model’s efficacy. The relevant changes have been incorporated into the Model development and performance evaluation section.

5. **In the feature selection process, the study involved a large number of feature variables. I am concerned about the potential for significant multicollinearity among these variables, which the researchers did not assess. The authors only used LASSO and PCA for dimensionality reduction.**

**Response：**We appreciate your insightful comment regarding multicollinearity. The use of LASSO regularization and PCA in our feature selection and dimensionality reduction process inherently addresses potential multicollinearity. LASSO naturally reduces the coefficients of correlated variables, while PCA transforms features into uncorrelated principal components, making the model robust to multicollinearity. While traditional multicollinearity diagnostics (such as VIF) were not conducted, we acknowledge that this could serve as an additional validation step in future studies. We will include this consideration in our discussion of limitations. Thank you for your valuable input.

6. **Given that the researchers identified data imbalance as an issue, experts in this field should recognize that the precision-recall curve can be more valuable than the ROC curve for evaluation.**

**Response:** Thank you for your insightful suggestion regarding the use of the precision-recall (PR) curve. We fully acknowledge that the PR curve can offer valuable insights, particularly in scenarios where data imbalance is significant and the focus is on the minority class. In our study, we addressed the imbalance issue by applying SMOTE and KNN to balance the distribution of our outcome variable before model development. This adjustment helped mitigate the imbalance and allowed us to use a variety of evaluation metrics, including AUC, sensitivity, specificity, PPV, and NPV, to comprehensively assess our model’s performance.

Given the clinical context of our research, where both false positives and false negatives carry significant weight, these additional metrics (sensitivity, specificity, PPV, and NPV) provided a more holistic evaluation, particularly for practical application in a clinical setting. While we recognize the merits of the PR curve, particularly in highly imbalanced datasets, we believe that our current evaluation metrics sufficiently capture the model’s performance after applying data balancing techniques. That being said, we are always open to refining our approach, and we appreciate your suggestion. We will certainly consider incorporating PR curves in future iterations of our analysis to further enhance the robustness of our model evaluation. Once again, we deeply value your feedback and remain committed to producing the highest quality work. Thank you for your thorough review.

7. **The methods used in this study are quite simplistic. With the availability of libraries like Sklearn and SHAP, the required code to complete this study likely amounts to less than 100 lines, indicating a lack of rigorous methodology in constructing the machine learning models.**

**Response：**Thank you for your insightful comments regarding the methodology and code structure of our study. We appreciate your observation about the streamlined nature of libraries like Sklearn and SHAP, which indeed facilitate concise and efficient machine learning implementations. However, we would like to clarify that the code initially uploaded with the submission pertains only to the web-based calculator, which was designed to be user-friendly and is, therefore, more concise. The actual code used for the machine learning model development, including feature engineering, handling data imbalance through SMOTE, hyperparameter tuning for seven models, and rigorous cross-validation, is significantly more extensive. These processes, especially the hyperparameter optimization, cross-validation, and detailed evaluation with multiple metrics (AUC, sensitivity, specificity, PPV, NPV), naturally result in a more complex and lengthy codebase.

Thus, while the uploaded calculator code may give the impression of a simplified approach, the full scope of our machine learning implementation reflects a high level of methodological rigor. We will be happy to provide the complete code used for the modeling process upon request to demonstrate the depth of our methodology.

We sincerely appreciate your feedback, which has provided us with an opportunity to clarify this point and further reflect on the importance of transparency and rigor in our study. Thank you again for your valuable suggestion!

**8.The web-based calculator is aesthetically unappealing. The data entered by each user should be visualized, for example, to highlight which specific indicators are most significant in determining high VTE risk. The authors used SHAP to improve model interpretability, so this should be reflected in the web-based calculator.**

**Response:** Thank you for your constructive feedback regarding the design of our web-based calculator. We sincerely apologize for the lack of visual appeal and the absence of data visualization, which could have better showcased the significance of individual indicators in determining VTE risk. Although we utilized SHAP to enhance model interpretability, we acknowledge that this was not adequately integrated into the calculator’s interface. In response to this, we will clearly mention in the Limitations section that the current version of the web-based tool does not visually display the SHAP-based interpretations. We will also indicate our plans for future improvements to incorporate data visualization elements, allowing users to interact with the results and gain insights into the contributions of specific variables to VTE risk prediction. We greatly appreciate your suggestion, as it highlights an important aspect of enhancing user experience and clinical utility, and we are committed to improving the design in subsequent iterations.

**Best regards,**

**Youli Jiang,**

**On behalf of all co-authors**
